# Catheterization

**Published:** 1899-12

**Authors:** L. A. Merillat

**Affiliations:** Secretary of M’killip Veterinary College, Chicago


### DEPARTMENT OF SURGERY.

By L. A. Merillat, V.S.,
SECRETARY OF M'KILLIP VETERINARY COLLEGE, CHICAGO.

ASSISTED BY

W. E. A. Wyman, M.D.V., V.S.,

MILWAUKEE, WIS.

J. A. Sloan, B.Sc., M.D.V.,

SAN FRANCISCO, CAL.

Dr. Robert Jay, M.D.V.,
WEST LIBERTY, IA.

### Catheterization.

The chief indications for catheterization in veterinary practice are:

*a.* Azoturia of the horse.

*b.* Parturient collapse of the ox.

*c.* Hypertrophied prostate of the dog.

There are, of course, a large number of other indications in which animals are unable voluntarily to void urine, or avoid doing so, on account of the pain produced by assuming the proper attitude for performing the act of micturition. Among these are fractures of the pelvis, pleurisy, painful wounds of the abdominal walls or extremities, rheumatism, etc., but the first-named constitute the more common diseases in which catheterization is an essential therapeutic factor. The veterinarian seldom finds it necessary to catheterize his patients—dog excepted—when they are able to maintain the standing posture long enough to urinate, for if an animal is made to suffer the slight pain of a distended bladder it will usually learn to perform the act voluntarily in due time. This is especially the case in fractures, pleuritis, and slight or unilateral azoturia. Therefore, catheterization is more frequently performed in the recumbent position and for those diseases in which this position dominates for a number of days.

The bladder may readily be emptied in both male and female horses by forcible pressure through the rectum or vagina, but as such a procedure causes more or less injury and straining the catheter is preferable. To perform the operation the patient is placed in the costal position, and is made to remain in this attitude by a strong assistant at the head. The first task that confronts the operator is to protract the penis. In doing so it is advisable not to lubricate the hands nor the sheath, for often the penis will become so slippery as to make its protraction quite troublesome. If the operator's hand is large and the patient's sheath is small the aperture of the latter can be dilated by having the uppermost leg elevated by an assistant. This simple step will make it possible to admit the largest hand into the smallest sheath. When the hand reaches the penis the thumb—palm forward—is hooked into the pouch above the meatus. The assistant at the head is now instructed to exert himself to prevent the patient regaining the sternal position, for as soon as traction is used considerable struggling may be expected. With the above-mentioned hold the penis is now pulled slowly but forcibly to the aperture of the sheath, and then it is grasped firmly, behind the glans, with the heretofore unoccupied hand and protracted as far as possible. The meatus and glans are then cleansed and anointed with carbolized vaseline. A well-lubricated and smooth catheter can then readily be passed into the bladder, there being resistance only as the instrument surmounts the ischium. In about 20 per cent. of all cases the unsuspecting will be astonished that the urine does not begin to flow, a circumstance that is quite as annoying to the experienced as it is surprising to the novice. In such cases the catheter has not reached the bladder at all but has passed into one of the ejaculatory ducts (some say the masculine uterus, and I have no evidence to offer to the contrary), and usually no ordinary amount of manipulating will revert it to the proper channel until the hand is placed into the rectum to direct its course. The operator now changes his position to the rear of the patient, holds the penis from between the legs with one hand and passes the other into the rectum. The assistant is now directed to manipulate the instrument backward and forward, while the hand within the rectum directs its extremity along the floor of the pelvis. A little patience will usually be rewarded, but the writer confesses failure in several instances.

The mare is catheterized by simply passing the well-lubricated hand into the vulva to direct the instrument and to lift the protecting valve of the meatus. The catheter is directed along the floor, and it readily finds its way to the bladder unhindered. The hand and instrument should be warm and thoroughly lubricated, to prevent straining. It is not good practice to empty the mare's bladder by pressure or even by dilating the meatus.

In the ox the male cannot be catheterized through the meatus on account of a well-known anatomical arrangement, and the female is catheterized with much more difficulty than the mare. The urethra terminates between a flexible fold, which is always the cause of some annoyance. The fold above mentioned is supported between the first and second finger of the left hand, while the right hand directs the instrument between them. In this way a little dexterous manipulation will accomplish the feat.

For catheterization of the dog, see Glass' translation of *Müller*.

As to the care of the veterinary catheter, it might be truthfully said that no implement in the veterinarian's paraphernalia is so shamefully cared for as the catheter, and, certainly, none requires better attention, both from a practical as well as an economical point of view. Being long, it is either thrown carelessly into the vehicle or else coiled up so as to admit it to the instrument-case or medicine-chest. In either case it is soon roughened and rendered unfit for insertion into so delicate a channel as the urethra. Coiling is particularly detrimental, in that it causes the polish to crack transversely throughout the whole length of the lesser curvature. When not in use a catheter should be encased in a chamois-bag and hung up from one extremity or laid straightened out upon a shelf or, what is still better, in a long wooden box, which also serves the purpose of protecting it when carried in a vehicle. On foot it can be conveniently carried around the body beneath one's outer clothing. The apparent ridiculousness of this latter suggestion is easily surpassed by its practicability.

The points to bear in mind in veterinary catheterization are:

1. Never catheterize more than once a day.

2. Never catheterize for more than four or five successive days. Rather encourage the patient to urinate alone.

3. Keep the catheter smooth, clean, disinfected, and well lubricated with a bland antiseptic ointment. A rough catheter is really a very dangerous instrument.

4. Always empty the rectum before operating, as it is quite impossible to pass the catheter when the rectum is full.

5. See to it that the assistant at the head is properly instructed in holding the patient in the costal position.

6. Operate by leaning over the patient and never take the dangerous position among the legs.

In defence of the first two statements, it might be mentioned that repeated catheterization, under conditions which confront the veterinary practitioner, is always followed by more or less severe urethritis, and often cystitis, or even nephritis.

L. A. MERILLAT.

---

### SURGICAL ITEMS.

*Another Plea for Better Surgery.* A flame of a match or a kettle of boiling water are two things so easily obtained that there is no excuse for using suspicious instruments or dressings. The writer remembers the days when a probe was taken out of the pocket-case and plunged into a wound with the utmost unconcern; when a suture was cut off from a quantity rolled up on a little piece of cardboard, and then, the end having been carefully pointed by rolling it between the finger and thumb, after the fashion of our dear old grandmothers, and dexterously pushed into the eye of the needle, was skilfully applied, with occasional nice crops of pus, laudable or otherwise. These were the good old days (for the microbes), and we are rather pleased that they have come and gone. Neglect in cleanliness is no longer allowable now that we know what the punishment is likely to be. The laity themselves have learned too much about asepsis not to feel very inquisitive when what they call blood-poisoning occurs as the result of the doctor's work, and will accept his excuses with bad grace. In fact, whenever anything is done which may possibly be followed by a septic result it will be wise to call the attention of the patient and his friends to the precaution taken against such a result. Then, if our efforts prove unavailing, they will be aware that we did all we could to avoid it. It is often on mighty little things that a man's reputation hangs, and an erysipelas after vaccination often hurts him more than a death after an amputation or a laparotomy.—*International Journal of Surgery.*

[While the above was written not for the veterinarian but as a warning to the human surgeon, the former can do no better than to read and weigh the force of each word carefully, and then endeavor to profit by this sensible plea. It strikes directly at a neglected and disregarded branch of our vocation.—L. A. M.]

There is no greater waste of money than that due to the habit of improperly caring for instruments, and the number that may be lost by neglect in the course of a year is surprisingly large. Before going out to an operation count your instruments, and count them again before returning them to your bag. When you wash your instruments rinse them in hot water and wipe them as they come out hot. They will dry much better. If the instrument is a complicated one and difficult to clean, put it in alcohol after wiping off as much of the water as possible. The alcohol will readily evaporate and leave a dry instrument. If the instruments are rusty boil them in carbonate of sodium solution and wipe them dry. Instruments which remain on the shelf should be wiped at least once a week. The nickel will then remain bright and keep its polish. Keep rubber instruments dusted with soapstone, to which a little carbonate of soda might be added. Soft rubber-instruments should not remain long in contact with each other, as they will have a tendency to adhere together and form rough spots. After using soft-rubber instruments alcohol will carry away moisture as well as disinfect them. Do not throw away rusty instruments. It is easier to have them sharpened and renickeled than to buy new ones. Spend a little time with your tools each week, for they will repay your care.—*Id.*

